# Biodegradation of Benzo(a)pyrene in Contaminated Soil: Plant and Microorganism Contributions from Isotope Tracing

**DOI:** 10.3390/toxics13050405

**Published:** 2025-05-16

**Authors:** Jianlong Wang, Xiaobing Su, Changhe Zhang, Zhimeng Han, Meiqi Wang

**Affiliations:** 1 Key Laboratory of Urban Storm Water System and Water Environment, Ministry of Education, Beijing University of Civil Engineering and Architecture, Beijing 100044, China; 2 Beijing Energy Conservation & Sustainable Urban and Rural Development Provincial and Ministry Co-Construction Collaboration Innovation Center, Beijing 100044, China; 3 China Academy of Building Research, Beijing 100013, China

**Keywords:** microbial degradation, plant absorption, volatilization, microbial community structure, isotope tracing

## Abstract

Biological degradation effectively removes benzo(a)pyrene (BaP) from contaminated soil; however, knowledge regarding the contributions of plant absorption, microbial degradation, and volatilization to BaP removal remains limited. In this study, the BaP removal pathway in contaminated soil was investigated. The structural evolution of the microbial community in contaminated soil was revealed using a comparative experimental study. BaP, as a representative of high-molecular-weight polycyclic aromatic hydrocarbons, was removed from freshly contaminated soil by microbial degradation, plant absorption, and volatilization in proportions of 20.955%, 12.771%, and 0.005%, respectively. The proportions of BaP removed by microbial degradation, plant absorption, and volatilization in aged contaminated soil were 29.471%, 16.453%, and 0.004%. Microbial degradation was the most responsible mechanism for BaP removal. Moreover, a higher number of BaP degrading bacteria occurred in the aged contaminated soil. At the genus level, *Pseudomonas* and *Sphingomonas* were detected in both types of soils, being the key bacterial species involved in BaP degradation.

## 1. Introduction

Polycyclic aromatic hydrocarbons (PAHs), typical persistent organic pollutants commonly found in contaminated soil [1], enter the environmental cycle mainly via the incomplete combustion of motor fuels, forest fires, and volcanic eruptions [2]. The primary remediation mechanisms of PAHs in polluted soil are volatilization [3], photodegradation [4], catalytic oxidation [5], soil adsorption [6], and biological absorption and degradation [7]. Biological absorption and degradation refer to the direct absorption of PAHs from polluted soil by plants or the conversion of PAHs into harmless substances through plant-microorganism co-metabolism [8], and the method has great advantages of high efficiency, low cost, and minimal secondary pollution [9].

Plants remove PAHs by exploiting two main mechanisms: first, plants directly uptake low molecular weight PAHs through absorption and cellular enzyme metabolism. These PAHs are then transported to the root, stem, and leaves, finally leaving the plant via leaf respiration [10]. The removal efficiency of PAHs from contaminated soil is positively correlated with plant biomass; more biomass allows for greater PAH absorption and removal [11]. Additionally, plant root exudates can significantly enhance the bioavailability of PAHs by increasing microbial diversity and their activity around the root [12,13]. Wang et al. evaluated the removal efficiency of PAHs in the Dagang oilfield soil using pot experiments [14]. After 150 days, PAH removal from the soil was primarily driven by stimulating rhizosphere microbial metabolism. Davin et al. investigated the role of root exudates of two legumes (*Medicago sativa* and *Trifolium pratense*) in improving the bioavailability and biodegradability of PAHs, reporting that the concentration of dehydrogenase, which enhances soil microbial activity, was significantly higher in the presence of these root exudates [15].

The microbial degradation of PAHs is influenced by several factors, such as microbial diversity, community structure, and the physical and chemical properties of soil [16]. PAHs can be removed by bacteria, fungi, and algae, where bacteria and fungi have superior PAH degradation abilities and are the most widely studied approaches [17]. Various microorganisms, including *Bacillus*, *Rhodococcus*, *Pseudomonas*, *Sphingomonas*, White rot fungi, and *Staphylococcus aureus*, were identified as microorganisms for PAH degradation [18]. *Bacillus licheniformis* and *Bacillus moharicensis* strains, which were isolated from petroleum-contaminated soil, effectively removed PAHs from the soil [19]. Yarahmadi et al. investigated the biodegradability of four isolated strains (*Pseudomonas*, *Mycobacterium*, *Rhodococcus*, and *Sphingomonas*) toward anthracene, phenanthrene, and pyrene removal and assessed the effects of environmental parameters, such as pH, temperature, nutrient content, and salinity, on their degradation efficiencies [20]. The optimal conditions for PAH removal were found to be a pH of 7.5, a temperature of 35 °C, and nitrogen and phosphorus concentrations of 5 and 2 g/L, respectively, and 1% salinity. Biodegradation experiments on PAH removal in polluted soil using *Aspergillus terreus* and *Penicillium* [21] were performed, and anthracene, phenanthrene, and pyrene were completely degraded after 68, 54, and 64 days, respectively. Overall, the reported findings prove that biodegradation is effective for PAH removal from polluted soil.

BaP is a highly toxic and strongly carcinogenic component of PAHs [22], commonly found in water, soil, automobile exhaust gases, and fossil fuel combustion products [23]. Although BaP can be removed from contaminated soil in different ways, the contribution of each of the pathways to the total BaP removal remains unclear. In this study, the BaP removal pathway from contaminated soil was investigated using ^13^C isotope tracing. Additionally, the temporal evolution of microbial community characteristics was investigated in BaP polluted soil using high throughput sequencing technology to provide insight into effective soil remediation strategies for organic pollutants.

## 2. Materials and Methods

### 2.1. Soil Preparation

Two types of soils were used for the experiments. The first soil type was sampled at a depth of 0–30 cm by a five-point sampling method. The sampling was performed at a PAH contaminated site located in Hangzhou, and the sample was denoted as the contaminated soil. The second soil type was sampled by the same method at farmland near the contaminated site; the sampling site itself was not polluted, but BaP was added during the experiment, and the sample was named the non-contaminated soil. The soil in this area mainly consists of clay mixed with small amounts of sand and limestone. Chemical analysis results reveal that the BaP concentration in the non-contaminated soil was below the detection limit, while in the contaminated soil, it was 0.42 mg/kg. The main pollutants in the contaminated soil sample were BaP, BaA, BbF, DahA, and total petroleum hydrocarbons. The additional physical and chemical properties of both soil types are shown in Table S1.

Since the concentration of BaP in non-contaminated soil was lower than the detection limit, exogenous BaP was added to evaluate the removal pathways of BaP in the two types of soils. A total of 75.51 mg BaP (≥96%, purchased from Shanghai Yien Chemical Technology Co., Ltd., Shanghai, China) was first dissolved in acetone (purchased from Shanghai Yien Chemical Technology Co., Ltd.), and 0.32 mg BaP-^13^C_16_ (purchased from Cambridge University, Cambridge, UK) was added. Subsequently, the BaP acetone solution was divided into two equal parts, and then each part was separately mixed with 200 g of soil (the one where the BaP concentration was below the detection limit). Each of the obtained soils was fully mixed with 5 kg of the non-contaminated soil (named freshly contaminated soil) and 5 kg of the contaminated soil (named aged contaminated soil), respectively. The mixtures were placed in a fume hood under dark conditions and aged for 48 h. The soil samples were then transferred into pots and aged for an additional 15 days before ryegrass was planted. The initial BaP concentrations in the freshly contaminated and aged contaminated soil were 6.91 and 7.07 mg/kg, respectively. During the experiment, the soil moisture content in the setup soil was maintained at approximately 60%, as determined using a gravimetric method.

### 2.2. Experimental Setups

**Figure 1:**
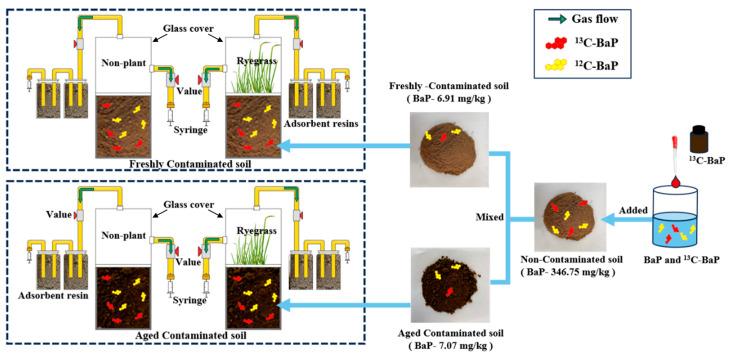
Experimental setups and soil preparation.

The experimental setups used in this study are illustrated in Figure 1. The diameter and height of the used ceramic pot were 20 and 25 cm, respectively, and a glass cover (with a diameter of 20 cm and a height of 35 cm) was placed on top to seal the system and facilitate gas collection. Four setups were used in the experiments (which contained two types of soils): freshly contaminated soil without plants (NCSN), freshly contaminated soil with ryegrass (NCSR), aged contaminated soil without plants (ACSN), and aged contaminated soil with ryegrass (ACSR). A gas exchange valve was attached to the glass cover, and two sealed bottles containing adsorption resins (DN-150) were connected to the glass cover to absorb volatile BaP. The sealed bottles were connected to a syringe. Soil, plants, gas in the setup, and the adsorption resins in the glass cover were sampled on Days 5, 10, 20, and 30, as well as at the beginning of the experiment. The adsorption resins were replaced with a new one after sampling. A 50 mL syringe was used to extract the gas from the glass cover, and the gas sample was stored in a 50 mL gas sampling bag.

### 2.3. Plant Selection

Ryegrass, with a height of approximately 30 cm, was used in the following experiment. It is a gramineous plant with good stress resistance, and it has been proven effective and widely utilized for BaP removal from contaminated soil [24,25]. Its root can absorb BaP from the soil, enhance microbial populations, and increase microbial diversity [26]. Moreover, ryegrass root exudates can also promote bacterial degradation activity in the soil, thereby strengthening the biological repair processes of microorganisms on BaP [27].

### 2.4. BaP and ^13^C Isotope Analytical Methods

#### 2.4.1. Determination of the BaP Concentration

An ultrasonic extraction method was used to isolate BaP from the soil based on a previous study by Guo and Wen [28]. After freeze-drying (FD-1A-50), milling, and passing through a 50-mesh sieve, 5 g of sample in a 50 mL centrifuge tube was added to 15 mL of dichloromethane and 15 mL of n-hexane (v:v = 1:1), and afterward, the mixture was ultrasonicated for 30 min. The suspension was centrifuged (DT5-2B) at 4000 rpm for 10 min, and the supernatant was transferred to a rotary evaporator (ER-52AA) after filtrating through a 0.22 μm organic membrane. These steps were repeated three times. The extracted substance was re-dissolved in 15 mL of acetonitrile after complete evaporation and stored at 4 °C for further analysis.

The BaP concentration was determined using a high-performance liquid chromatograph (Agilent 1260). The chromatographic column was an Eclipse PAHs (250 × 4.6 mm, 5 μm): 959990-918. Acetonitrile and deionized water were used as the mobile phase (85%:15%), and the sample volume was 10 μL. The external standard method was employed to quantify BaP, with the standard curve R^2^ value of 0.9999 and a recovery rate ranging from 89.3 to 106.8%.

The removal rate of BaP in soil was calculated using Equation (1):

(1) $$V_{i}=\frac{C_{0}-C_{1}}{C_{0}}$$

where *V_i_* is the removal rate of BaP in the soil (%), *C*_0_ is the initial concentration of BaP in the soil (mg/kg), and *C*_1_ is the remaining BaP concentration in the soil (mg/kg).

#### 2.4.2. Calculation of the Relative Isotope Abundance

As natural isotopes exist in nature, more attention in practical research is paid to the relative changes in the isotope ratio rather than the absolute isotope ratios. Thus, the specific isotope composition is commonly represented using the δ isotope value, representing the relative difference between the ratio of two isotopes in the investigated sample and a specific standard. The two most stable carbon isotopes are ^12^C and ^13^C, with relative abundances of 98.89 and 1.11%, respectively. The δ^13^C ratio was calculated using the Vienna Pee Dee Belemnite (VPDB) standard, a widely used reference established by a laboratory in Vienna [29], as given in Equation (2):

(2) δ13C Value‰=R(C13/Csample12)R(C13/CVPDB12)−1×1000

where *R* (^13^*C/*^12^*C_VPDB_*) is the carbon isotope abundance ratio of VPDB, and *R* (^13^*C/*^12^*C_sample_*) is the carbon isotope abundance ratio of the investigated sample.

The ^13^C isotope abundance of solid samples was measured using Sercon Integra-2 elemental analysis (Sercon Co., Ltd., Crewe, UK) for isotope-ratio mass spectrometry. Standard substances in the test included IAEA-600 (δ^13^C = −27.71‰), uSGS-40 (δ^13^C = −26.39‰), and a laboratory internal standard for acetanilide (δ^13^C = −26.85‰). The solid sample was freeze-dried for 48 h, ground, and passed through a 100-mesh sieve. The sample was wrapped in aluminum foil for testing.

The temperature in the oxidation tube, reduction tube, and gas chromatography column was set to 1000, 640, and 50 °C, respectively. The He pressure was 29 psi, the He flow rate was 18 mL/min, and the O_2_ pressure was 30 psi. Four blanks were set before the sample test, and two standard samples were inserted for each of the 12 samples.

The concentration of CO_2_ and the abundance of the ^13^C isotope were measured using a Delta V Advantage isotope mass spectrometer (Thermo Scientific Co., Ltd., Waltham, MA, USA). A CTC Analytics CombiPAL automatic sampler was applied, while headspace sampling was employed for injection. The chromatographic column was an Agilent PoraPlotQ (30 m × 0.32 mm × 20 μm).

After removing the air present in the sample bottle, the collected 100 μL gas samples were extracted using an automatic sampler and transferred to an injection bottle for testing. The gaseous samples were injected by an automatic injection method, the carrier gas was a mixture of He and CO_2_, and the column temperature was 75 °C. Different volumes of standard CO_2_ gases (>99.99%) were tested to produce a standard curve (R^2^ = 0.9999).

The analysis error of the solid sample was ±0.10‰, while that of the gas sample was ±0.2‰. The initial δ^13^C value in the non-contaminated soil (before adding BaP) was 1.095 atmo.%; in the contaminated soil (before adding BaP), it was 1.092 atmo.%; and in the ryegrass, it was 1.077 atmo.%. The initial δ^13^C-CO_2_ value in the glass cover was −14.49‰.

### 2.5. High-Throughput Sequencing

Non-culture-based molecular biology methods enable rapid and systematic analyses of the microbial composition, structure, and diversity in environmental samples. Woese and Fox analyzed the phylogenetic relationship of prokaryotes based on the 16S rRNA gene sequence and proposed the famous three-domain theory [30]. Since then, the 16S rRNA gene sequence has become the most commonly used biomarker for studying microbial phylogeny, classification, and diversity. Herein, we employed a high-throughput sequencing technology to detect the 16S rDNA/rRNA gene in soil microbial cells. These genetic markers exhibit a certain degree of evolutionary conservation, and the conserved regions are common to all similar microorganisms and variable regions arising from evolutionary processes [31]. Therefore, the diversity of microbial species and the community structure in the soil can be unraveled by measuring and comparing these sequence-variable regions.

DNA extraction was performed following the protocol proposed by Cao et al. [32]. Soil DNA was quantitatively extracted from soil samples using 0.25 g of each sample by an E.Z.N.A.^®^ soil DNA kit (Omega Bio-tek, Norcross, GA, USA), according to the manufacturer’s instructions, and detected by 2% agarose gel electrophoresis. The extracted DNA was stored at −20 °C. The PCR amplification primers were 338F (5′-ACTCCTACGGGAGGCAGCAG-3′) and 806R (5′-GGACTACNNGGGTACTAAT-3′). Third-generation sequencing was performed, Q5 high-fidelity DNA polymerase provided by NEB was used for PCR amplification, and a control group was set. The above PCR amplification products were detected using 2% agarose gel electrophoresis. The sequencing library was prepared using PacBio’s Template Prep Kit 1.0 reagent, and the samples were sequenced using a PacBio Sequel sequencer.

## 3. Results and Discussion

### 3.1. Temporal Evolution of the BaP Concentration

**Figure 2:**
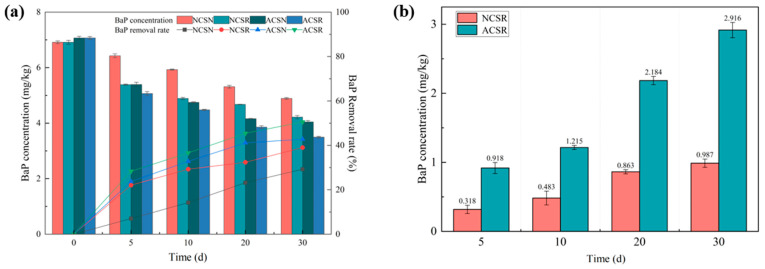
Temporal changes in the BaP concentration in soil for different experimental setups: (**a**) the BaP concentration and removal rate, (**b**) the BaP concentration in ryegrass in NCSR and ACSR setups.

The temporal changes in the BaP concentration in the soil for the four experimental setups (NCSR, NCSN, ASCR, and ACSN) are shown in Figure 2a. On Day 30, the residual concentration of BaP in NCSN, NCSR, ACSN, and ACSR soils was 4.89, 4.22, 4.05, and 3.50 mg/kg, respectively. The decrease rate of the BaP concentration within 30 days in the different experimental setups followed the order of ACSR (0.119 mg·kg^−1^·d^−1^), ACSN (0.101 mg·kg^−1^·d^−1^), NCSR (0.090 mg·kg^−1^·d^−1^), and NCSN (0.067 mg·kg^−1^·d^−1^). The BaP degradation rate in ACSR from Day 0 to Day 5 was 0.400 mg·kg^−1^·d^−1^, which is higher than the 0.036 mg·kg^−1^·d^−1^ from Day 5 to Day 30 (*p* < 0.01). The BaP concentration decrease rate was high in the early stage of the experiment due to the high amount of BaP in the soil available for utilization and uptake.

Regarding the experimental setups with the freshly contaminated soil, the BaP concentration in NCSR on Day 30 was 0.67 mg/kg lower than that in NCSN, and the average removal rate in NCSR on the same day was 0.023 mg·kg^−1^·d^−1^ higher than that in NCSN (*p* < 0.05). Similarly, after planting ryegrass, the BaP concentration in ACSR on Day 30 was 0.55 mg/kg lower than that in ACSN, while the average BaP concentration degradation rate in ACSR on the same day was 0.018 mg·kg^−1^·d^−1^ higher than that in ACSN (*p* < 0.05). The BaP removal rates in NCSR and ACSR were 39.000% and 50.553%, respectively, higher than those of the experimental setups without the plants (*p* < 0.01). Thus, the results confirm that plants successfully improve the removal BaP rate. In the phytoremediation process (e.g., by ryegrass), the interaction between plant root exudates and soil microorganisms may be enhanced, rendering the microbial degradation of PAHs in soil easier. The established plant–microorganism network can more efficiently remediate pollutants in case the soil again becomes contaminated by PAHs [33].

On Day 30, the BaP removal rates in ACSR and ACSN were 3.77% and 4.58%, respectively, higher than those in the freshly contaminated soil sample. Due to the long-term BaP pollution of the aged contaminated soil sample, the microbial community changed, and the abundance of BaP-degrading bacteria and the enzymatic activity in the soil were higher, resulting in a higher BaP removal efficiency in the aged contaminated soil sample [34,35]. It was shown that the diversity of bacterial communities in soil significantly changes after oil spills, e.g., the number of hydrocarbon-degrading genes rapidly increases, and the abundance of TPH degrading genes increases by 2–7 times [36]. The microbial community in the soil undergoes a certain adaptation and screening process upon first exposure to PAH pollution, so the PAH degrading microorganisms gradually become dominant. In the case of repeated soil contamination by the same type of PAHs, these adapted microorganisms can respond more quickly, thereby improving the PAH removal efficiency [37]. Moreover, the activity of some pollutant degrading enzymes (e.g., polycyclic aromatic hydrocarbon monooxygenase) in the soil may be increased, remaining at a high level for a certain time. When a similar re-contamination occurs, these enzymes rapidly respond and degrade new pollutants, thereby accelerating PAH degradation [38].

The BaP concentration in ryegrass, Figure 2b, increases with time, indicating an efficient absorption of PAHs by ryegrass [39]. The BaP concentration of ryegrass in ACSR was 1.928–0.600 mg/kg during the entire experiment, higher than that in NCSR (*p* < 0.05). On Day 30, the BaP concentration in ACSR was 2.916 mg/kg, higher than that in NCSR (0.987 mg/kg) (*p* < 0.05). This trend could be explained by the high content of organic matter in the aged contaminated soil, plausibly providing sufficient nutrients for plant growth, resulting in a higher BaP absorption efficiency [40]. A certain BaP amount may remain in the soil due to previous pollution. In the absence of plants, these residual substances can accumulate with new pollutants over time. As plants grow, they absorb both residual and new pollutants, increasing the BaP concentration within the plant. After contamination, the plant may undergo physiological and metabolic adaptive changes, making the BaP absorption more effective. When re-exposed to the same or similar contaminated environments, plants may show higher absorption efficiency due to the previous overall adjustments [41].

### 3.2. Temporal Evolution in the ^13^C Isotope Abundance

#### 3.2.1. ^13^C Isotope Abundance in Air

**Figure 3:**
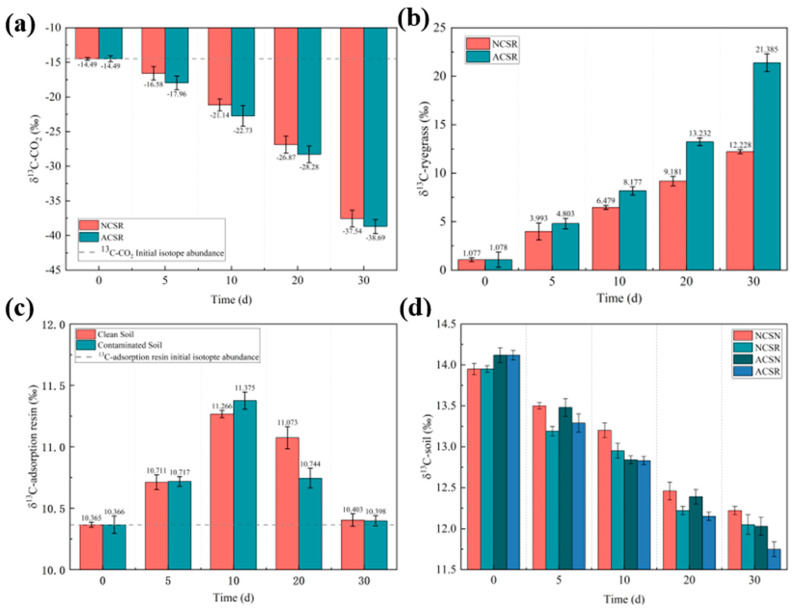
Temporal changes in the ^13^C-BaP value: (**a**) the δ ^13^C value of air in the experimental setups, (**b**) the δ ^13^C value in ryegrass, (**c**) the δ^13^C value in the adsorption resins, (**d**) the δ^13^C value of soil.

The initial CO_2_ concentration and the initial δ^13^C-CO_2_ value of air in the experimental setups were 0.08 mmol/L and −14.49‰, respectively. Figure 3a shows the changes in the δ^13^C-CO_2_ values in NCSR and ACSR with time. The δ^13^C-CO_2_ in the glass cover decreased from −14.49‰ at the beginning of the experiment to −38.69‰ on Day 30 in ACSR (*p* < 0.05). Moreover, the δ^13^C-CO_2_ in the glass cover decreased to −37.54‰ on Day 30 in NCSR (*p* < 0.05), possibly due to the higher concentration of organic matter in the aged contaminated soil and stronger photosynthesis of ryegrass, resulting in more ^13^CO_2_ being absorbed in the air.

#### 3.2.2. ^13^C Isotope Abundance in Plants

The δ^13^C value of ryegrass for NCSR and ACSR, Figure 3b, increases with time. In NCSR, the δ^13^C value of ryegrass increases from 1.077 to 12.228‰ (*p* < 0.01), while in ACSR, it increases from 1.078 to 21.385‰ (*p* < 0.01), indicating a successful uptake of BaP from the soil by plants [42]. Moreover, the δ^13^C value of ryegrass in ACSR was consistently higher than in NCSR during the entire experiment, similar to the trend found in Section 3.1, in which the BaP concentration of ryegrass in NCSR and ACSR increased, while the BaP concentration in ryegrass in ACSR was constantly higher than that in NCSR. These findings could be explained by the better resistance to BaP of ryegrass planted in the aged contaminated soil, allowing a higher uptake of BaP from the soil [43].

#### 3.2.3. ^13^C Isotope Abundance of the Adsorption Resins

The BaP concentration of the adsorption resins in NCSR and ACSR was lower than the detection limit, while the δ^13^C value for these samples is shown in Figure 3c. The initial δ^13^C value was 10.365‰. The δ^13^C value of the adsorption resins in NSCR and ASCR increased by 9.000 and 10.094‰, respectively, from Day 0 to Day 10, and then decreased by 8.625 and 9.773% to Day 30 (*p* < 0.05), respectively, indicating that BaP in the early experimental stage exhibited greater volatility. In addition, the average temperature was 10 °C, which could contribute to the BaP volatilization inhibition [44].

#### 3.2.4. ^13^C Isotope Abundance in Soil

The temporal evolution of the δ^13^C value in different experimental setups is shown in Figure 3d. The initial δ^13^C values in the aged contaminated soil and freshly contaminated soil were 14.117 and 13.949‰, respectively, while on Day 30, the δ^13^C values of the soil in NCSN, NCSR, ACSN, and ACSR were 12.221, 12.045, 12.034, and 11.746‰, respectively, and they decreased with time as follows: in NCSR, the δ^13^C value decreased by 1.904‰, in NCSN by 1.727‰; in ACSR, it decreased by 2.371‰; and in ACSN, it decreased by 2.083‰ (*p* < 0.05). Therefore, planting ryegrass promoted the BaP removal from the soil [26]. Compared with NSCN, the δ^13^C value in ASCN was 0.187‰ lower on Day 30. Similarly, the δ^13^C value in ACSR on Day 30 was lower compared with that in NSCR, indicating that the BaP removal efficiency in the aged contaminated soil was higher than in the freshly contaminated soil after the BaP pollution event (*p* < 0.01), which is consistent with the phenomenon presented in Section 3.1.

### 3.3. Contributions of ^13^C-BaP Removal in Soil

The degradation pathways of ^13^C-BaP for ACSR are listed in Table 1. The proportion of BaP in the soil of ACSR decreased with time, while the proportions of BaP absorbed by plants and degraded by microorganisms were 16.453 and 29.471%, respectively, on Day 30, indicating a higher contribution by microorganisms than by plants. A similar ^13^C-BaP degradation pathway was observed in NCSR, as shown in Table 2. The proportions of BaP absorbed by plants and degraded by microorganisms on Day 30 were 12.771 and 20.955%, respectively. Plants process pollutants through absorption, transport, and metabolism, but their efficiency is usually limited to a part of BaP and PAHs in general. Plants are more significant in creating a suitable environment for microbial growth around the root, indirectly enhancing microbial degradation efficiency [45]. Microorganisms can directly decompose and mineralize BaP and PAHs, transforming these organic pollutants into simple inorganic molecules (such as carbon dioxide and water) or less toxic intermediates. Compared to OECD/ISO 307 guideline-based biodegradation tests, which are widely adopted in the scientific community [46], the microbial degradation rates observed in this study align with reported ranges for BaP (21.6%) [47]. Specific bacteria and fungi can effectively metabolically degrade these complex organic molecules. However, high concentrations of BaP may destroy the integrity of microbial cell membranes and inhibit enzyme activity and metabolic function [46], and microorganisms need to adapt to high concentrations of pressure for a longer time.

Furthermore, the proportion of BaP removed by volatilization in the two experimental setups was less than 1% because BaP is a high-ring PAH, and it hardly volatilizes, so volatilization is not the main BaP removal pathway from the soil [48]. The BaP removal rate in ACSR was 45.93%, higher than 36.73% in NCSR on Day 30 (*p* < 0.01). The proportions of ^13^C-BaP absorbed by ryegrass and degraded by microorganisms in ACSR were 16.4533.68 and 29.471%, higher than 12.771 and 20.955% in NCSR (*p* < 0.05), respectively, ultimately making the BaP removal rate in ACSR higher than in NCSR. Indigenous microorganisms in the soil adapt to long-term pollution by enhancing their BaP degradation capacity [49]. Microorganisms play a pivotal role in bioremediation, contributing approximately 20–30% to the total remediation effect. They degrade and absorb pollutants directly through metabolic pathways, which makes microbial remediation an essential strategy within bioremediation. To enhance the PAH degradation, and in particular, the BaP degradation capacity of microorganisms, it is essential to select and utilize highly effective strains. Introducing such efficient strains into contaminated soil or adding nutrients to stimulate the activity of native microbial communities can significantly enhance the degradation efficiency of pollutants [50]. While phytoremediation directly accounts for only 10% of the remediation effect, it substantially supports microbial growth by generating root exudates, such as organic acids and sugars, which, in turn, indirectly enhance microbial degradation capabilities. Cultivating efficient microbial communities within the plant rhizosphere can further improve the overall remediation efficiency.

### 3.4. Evolution Characteristics of the Microbial Community Structure

#### 3.4.1. Taxonomic Annotation of Species

For the 16S rRNA gene sequence of bacteria, we used the classification sklearn algorithm of QIIME2 to annotate the representative sequences of each Operational Taxonomic Unit (OTU) [51], and the results are shown in Table 3 (*p* < 0.05).

From the beginning to Day 15, the number of phyla and classes in NCSR increased, while the number of families and genera decreased, suggesting that the abundance of some bacteria in NCSR increased, plausibly via the emergence of new bacterial species. However, at the family and genus level, specific bacteria likely began to dominate, reducing bacterial diversity. Meanwhile, in ACSR, the number of phyla, classes, orders, and families increased, while the number of genera and species decreased. This change might be due to the expansion of dominant bacterial populations in the soil, suppressing the growth of other bacteria. By Day 30, the number of phyla, classes, and orders in NCSR decreased compared to Day 15, and the number of families and genera remained relatively unchanged, while the number of species decreased. This suggests that the microbial community in the NCSR sample became more stable with time, where certain groups began to outnumber others in the community structure. The determined numbers for each classification level in the NSCR soil were higher than those in the ASCR soil because the contaminated soil was previously contaminated with PAHs, resulting in its poorer microbial diversity [52].

#### 3.4.2. Diversity of the Soil Bacterial Community

The bacterial diversity indices are listed in Table 4 (*p* < 0.05). The Chao1 index of NCSR was 1836.98 on Day 30, while that of ACSR was 1502.24, indicating a higher bacterial abundance in NCSR. After adding BaP, the Chao1 index in NSCR decreased from 2888.52 at the beginning of the experiment to 1836.98 on Day 30. Meanwhile, the Chao1 index in ASCR decreased from the initial value of 1539.23 to 1318.93 on Day 15, after which it increased again to 1502.24 on Day 30. The Chao1 index in NSCR and ASCR decreased by 1051.54 and 220.30, respectively. Microbial communities in ASCR soils showed greater resilience to BaP contamination, while microbial communities in NSCR exhibited a significant decay in diversity and abundance after contamination, suggesting that microorganisms in chronically contaminated soil may have improved their tolerance to pollutants through adaptive evolution or other mechanisms [53]. In addition, the observed species and the Shannon index showed similar trends to the Chao1 index. The Shannon index in the freshly contaminated soil decreased from 11.23 to 9.07 on Day 30, and the Shannon index in the ASCR soil decreased from 6.86 to 6.06 on Day 15 and then increased to 7.02 on Day 30. The number of observed species in NSCR decreased from 3043.9 to 1575.70 on Day 30, while it decreased in ASCR from 694.36 to 542.55 on Day 15 and then increased to 675.47 on Day 30. Thus, within 30 days, the microbial richness of NSCR continued to decrease, whereas the microbial richness of the ASCR decreased at the initial stage and then increased to the initial number. The changes in the diversity index indicate that the bacterial species diversity in NSCR was higher than in ASCR.

**Figure 4:**
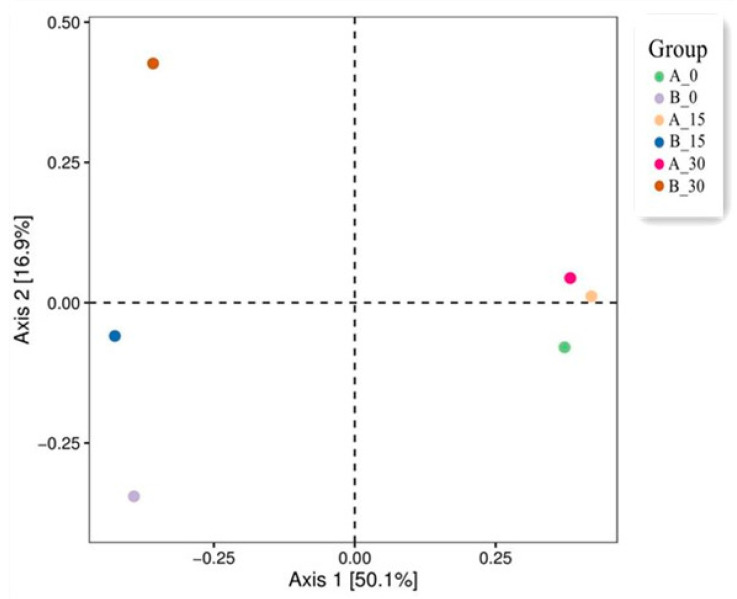
A two-dimensional sorting diagram of the biological communities in soil samples from the PCoA analysis.

Figure 4 shows a two-dimensional ordination diagram from the principal coordinates analysis (PCoA) of the biological communities in soil samples. The interpretation rate of Axis 1 (the *x*-axis) was 50.1%, while that of Axis 2 (the *y*-axis) was 16.9%. A significant difference in the composition of bacterial communities between NSCR and ASCR appeared. The distribution of sampling points in each NSCR group on Days 0, 15, and 30 is concentrated in the graph, and the community structure in NSCR was not considerably different. In contrast, the distribution of sampling points in the ASCR group was more dispersed, suggesting that the BaP addition significantly influenced the bacterial community in the aged contaminated soil, resulting in a distinct community structure in ASCR [54].

#### 3.4.3. Structural Composition of the Soil Bacterial Community

**Figure 5:**
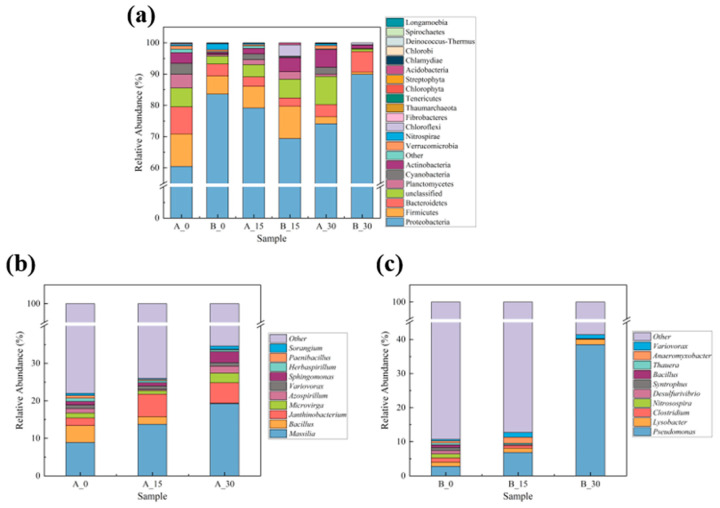
Composition of the soil bacterial community structure. Relative abundances of microorganisms: (**a**) for different soil phyla, (**b**) at the genus level in NCSR (TOP10), and (**c**) at the genus level in ACSR (TOP10).

To further assess changes in the soil microbial abundance during the experiment, the number of corresponding OTUs was counted at the phylum and genus levels. The bacterial species classifications of NSCR and ASCR substantially changed. Figure 5a shows the relative abundance of microorganisms across various soil phyla. At the beginning of the experiment, the NSCR group mainly included Proteobacteria, Firmicutes, Bacteroidetes, Planctomycetes, Cyanobacteria, Actinobacteria, Verrucomicrobia, and Nitrospirae bacterial species at the phylum level, while the ASCR group mainly contained Proteobacteria, Firmicutes, Actinobacteria, Bacteroidetes, Planctomycetes, and Verrucomicrobia, demonstrating a little compositional difference in the phylum-level bacterial communities between NCSR and ACSR (*p* < 0.05). Unlike NSCR, ASCR did not contain Cyanobacteria, which are typically dominant in oxygen-rich environments. Their absence may indicate changes in redox conditions within contaminated soils, or a decline of these microorganisms was caused by pollutant toxicity [55].

The relative abundance of Proteobacteria, the most abundant phylum in NSCR, increased from 60.46% before the experiment to 74.11% on Day 30. In contrast, the relative abundance of Cyanobacteria decreased from 3.53 to 2.15% during the same period. Similarly, the relative abundance of Actinobacteria increased from 3.40 to 5.65% by Day 30 of culturing, while the abundance of Nitrospirae increased from 0.46 to 0.61%. These changes indicate that these bacteria were resistant to pollutants, which is consistent with the findings of Liu et al. [56]. The relative abundance of Proteobacteria was the highest in ASCR, and it decreased from 83.59% on Day 0 to 69.38% on Day 15, after which it rose again to 89.93% on Day 30. The relative abundances of Actinobacteria, Bacteroidetes, and Chloroflexi increased from the beginning of the experiment to Day 30 from 0.51 to 0.99%, from 3.81 to 6.57%, and from 0.18 to 0.64%, respectively. The relative abundances of Firmicutes, Planctomycetes, and Verrucomicrobia decreased with time. Proteobacteria and Actinobacteria can degrade PAHs [57,58], and Bacteroidetes, along with Chloroflexi, are known for their BaP degradation abilities [59,60].

Microorganisms with the highest relative abundance at the genus level in NCSR (TOP10) are shown in Figure 5b. The relative abundances of *Massilia*, *Janthinobacterium*, *Sphingomonas*, *Microvirga*, *Azospirillum*, and *Sorangium* in NSCR increased compared with Day 0. *Massilia*, a potential PAH degrading bacterium, exhibits a robust degradation ability across various environmental conditions [57,61]. It increased from 8.90% (Day 0) to 19.25% (Day 30). *Janthinobacterium*, which also demonstrates the potential for PAH degradation, especially under specific environmental conditions, increased from 9.70% (Day 0) to 21.55% (Day 30). *Sphingomonas*, one of the microorganisms most involved in PAH degradation, is also frequently detected in PAH contaminated soil and has broad application prospects [17,62]. Its relative abundance increased from 0.92% (Day 0) to 2.89% (Day 30). Zhou et al. [63] demonstrated that *Sphingomonas* is not only able to degrade PAHs but also remains dominant at coking sites due to its metabolic capabilities and mobility, facilitated by chemotaxis. Finally, the relative abundances of *Bacillus*, *Variovorax*, *Paenibacillus*, and *Herbaspirillum* decreased as time elapsed.

Microorganisms with the highest relative abundance at the genus level in ACSR (TOP10) are shown in Figure 5c. The relative abundances of *Pseudomonas*, *Lysobacter*, and *Variovorax* in ASCR increased. The relative abundances of *Clostridium*, *Nitrosospira*, *Desulfurivibrio*, *Syntrophus*, *Bacillus*, *Thauera*, and *Anaeromyxobacter* decreased over the same experimental period. Among these, the relative abundances of *Pseudomonas* increased from 2.79% (Day 0) to 38.5% (Day 30). The effective degradation of PAHs in soil by *Pseudomonas* has already been demonstrated. Bhatawadekar et al. [64] successfully isolated bacterial strains from PAH contaminated river sediments, particularly noting the strong anaerobic degradation capabilities of *Pseudomonas sp*. for PAHs. Although there are relatively few studies on the direct degradation of PAHs or BaP by *Lysobacter*, its rich secondary metabolites and enzyme systems are promising for pollutant degradation [65]. *Lysobacter* can indirectly support other microorganisms in PAH degradation by secreting enzymes or metabolic intermediates. *Variovorax*, which increased from 0.46% (Day 0) to 1.176% (Day 30), can process the intermediate products of complex organic compounds, including PAHs, thus promoting an efficient degradation process [66].

The change in the microbial abundance in the soil can be correlated to the change in the BaP concentration. As the abundance of BaP-degrading microorganisms increased, the BaP concentration decreased (*p* < 0.05).

Figure 5c demonstrates significant differences in the bacterial genera between NSCR and ASCR, with a marked variance in the relative abundances of each genus (*p* < 0.05). Compared with NSCR, new dominant bacteria, such as *Pseudomonas*, *Lysobacter*, *Clostridium*, *Nitrosospira*, *Desulfurivibrio*, *Syntrophus*, *Thauera*, and *Anaeromyxobacter*, were found in ASCR. In general, in PAH contaminated soils, the microbial community undergoes a process of selection and adaptation, enhancing its ability to degrade PAHs and developing a greater resilience to further contamination. As a result, these communities respond quickly when re-exposed to pollutants. In contrast, microbial communities in non-contaminated soil fail to develop this adaptability, requiring a longer adjustment period for their community structure, which leads to significant differences in microbial composition [67].

**Figure 6:**
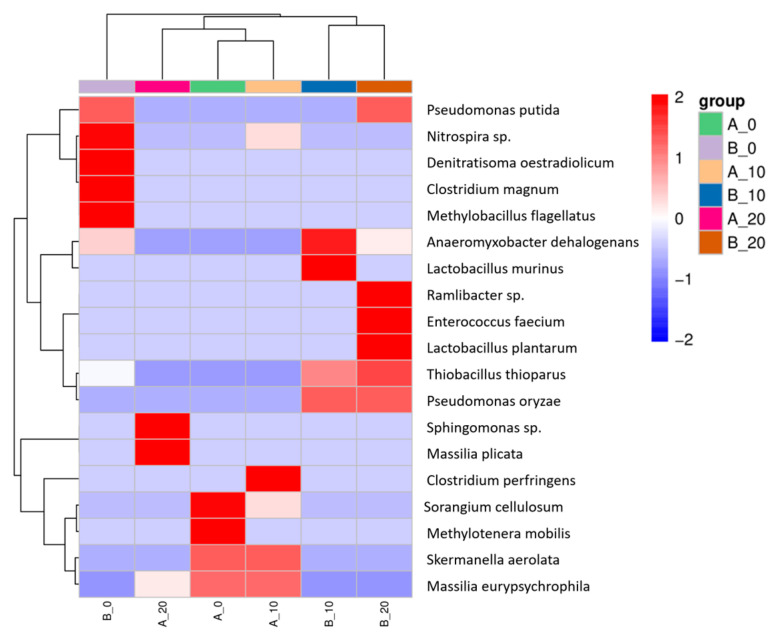
Species-level clustering.

The number of bacterial OTUs at the species level was analyzed, and the top 50 bacterial species based on the average abundance were selected to draw a cluster heatmap, as shown in Figure 6. The colors in the heatmap represent species abundance. On Day 30, the relative abundances of *Sphingomonas* sp. and *Massilia plicata* in NSCR increased, while those of *Sorangium cellulosum* and *Methyliotenera mobilis* decreased compared to the respective initial levels. The relative abundances of *Ramlibacter* sp., *Enterococcus faecium*, and *Lactobacillus plantarum* were higher in ASCR than in NSCR, indicating that the former environment is rich in organic matter or generates specific ecological conditions that support the proliferation and activity of these bacteria. Moreover, the relative abundance of *Pseudomonas oryzae* in ASCR was higher on Days 15 and 30. Among these, *Pseudomonas putida* and *Sphingomonas* sp. are known environmental pollutant degrading bacteria, particularly effective in degrading PAHs, such as BaP [68], so the high abundance of *Pseudomonas putida* in sample B_0 indicates the presence of pollutants in this environment.

**Figure 7:**
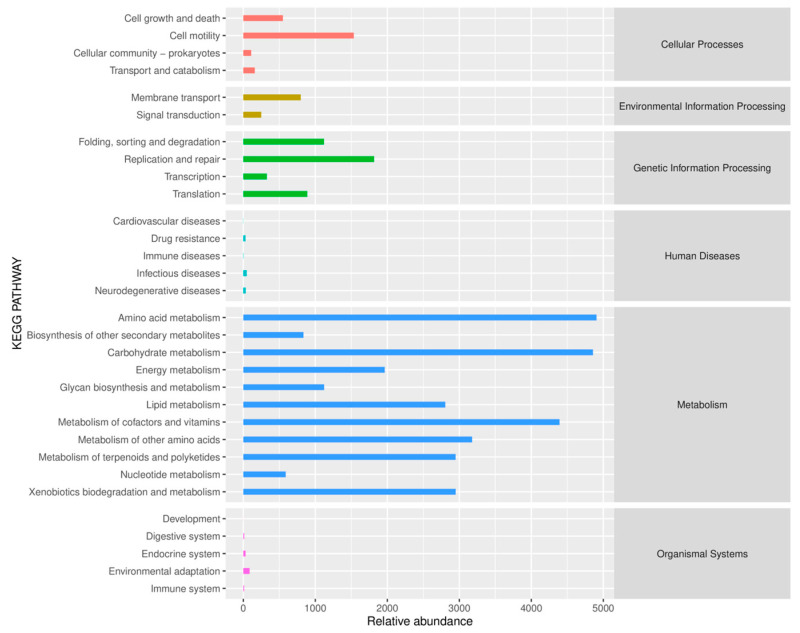
The predicted KEGG secondary functional pathway abundance map of microorganisms in ACSR.

KEGG functional annotation analysis reveals characteristic metabolic features associated with benzo[a]pyrene (BaP) degradation in soil microbial communities under BaP contamination, as shown in Figure 7. The result of ACSR demonstrates significant activation of BaP specific xenobiotic biodegradation pathways, potentially involving specialized membrane transport systems for BaP translocation. Concurrently, vigorous carbohydrate and energy metabolism systems supplied both energetic provisions to sustain BaP oxygenation and aromatic ring cleavage [69]. The abundance of repair and membrane transport was higher, which reflected the adaptive response of microorganisms to pollutant stress. This systematic coordination of functional modules suggests a multi-component synergistic mechanism enabling efficient biotransformation and mineralization of BaP [70].

## 4. Conclusions

In this study, we comprehensively addressed the pathways of BaP removal from soil by carefully designing our experimental approach. Exogenous BaP was added to simulate the pollution events in contaminated soil and non-contaminated soil samples. ^13^C isotope tracers were used to assess the pathway of BaP removal from contaminated soils. The evolutionary characteristics of microbial communities over time were investigated using a high-throughput sequencing technology.

Microbial degradation and plant absorption were the main pathways for removing BaP from the investigated soil samples. Planting ryegrass was demonstrated to be an effective method for removing BaP from the contaminated soil, but microbial degradation was the most significant contributor to BaP removal, with higher proportions observed in the aged contaminated soil. The BaP removal rate in the aged contaminated soil was higher than in the freshly contaminated soil when both types of soils were exogenously contaminated by BaP. In the aged contaminated soil, microbial communities adapted over time to the presence of BaP and other contaminants, so they degraded BaP more efficiently than those microorganisms in the freshly contaminated soil. After BaP pollution, the richness of microbial species in the aged contaminated soil recovered faster and its resilience was stronger compared to the freshly contaminated soil, whose species richness decreased continuously. According to the PCoA analysis of biological communities, the addition of BaP caused a greater change in the microbial structure of the freshly contaminated soil. At the genus level, *Pseudomonas* and *Sphingomonas*, the key bacterial species in the degradation of PAHs, were detected in both freshly contaminated and aged contaminated soils. To enhance the microbial degradation capacity, it is important to select and utilize microbial strains that can effectively degrade PAHs, and in particular, BaP. At the same time, the microbial degradation efficiency can also be enhanced through phytoremediation.

## Figures and Tables

**Table 1:** Contributions of ^13^C-BaP removal in ACSR.

Time (d)	Soil Residue (%)	Uptake by Plants (%)	Microbiological Degradation (%)	Volatilization (%)
5	87.793	2.331	9.836	0.044
10	76.352	4.441	17.081	0.126
20	63.022	11.604	25.327	0.047
30	54.072	16.453	29.471	0.004

**Table 2:** Contributions of ^13^C-BaP removal in NCSR.

Time (d)	Soil Residue (%)	Uptake by Plants (%)	Microbiological Degradation (%)	Volatilization (%)
5	92.262	2.344	5.348	0.046
10	81.278	4.269	14.333	0.120
20	67.098	8.073	18.735	0.094
30	63.269	12.771	20.955	0.005

**Table 3:** Identification results of microbiological taxonomic notes.

Sample	Phylum	Class	Order	Family	Genus	Species
A_0	17	28	41	181	154	7
B_0	4	48	18	70	59	8
A_15	19	10	23	139	130	7
B_15	14	34	16	56	48	6
A_30	10	15	29	134	103	3
B_30	18	16	2	27	134	12

A_0, A_15, and A_30 represent the soil in NCSR sampled on Day 0, 15, and 30, respectively; Day 0 was the beginning of the experiment; B_0, B_15, and B_30 represent the soil in ACSR sampled on Day 0, 15, and 30, respectively.

**Table 4:** Microbial diversity index.

Sample ID	Chao1 Index	Simpson	Shannon	Pielou’s Evenness	Observed Species	Faith’s PD	Good’s Coverage
A_0	2888.52	0.99	11.23	0.97	3043.91	306.98	0.94
A_15	2269.22	0.99	10.52	0.98	2499.62	281.13	0.88
A_30	1836.98	0.98	9.07	0.85	1575.70	198.67	0.97
B_0	1539.23	0.96	6.86	0.73	694.36	57.46	0. 89
B_15	1318.93	0.97	6.06	0.78	542.55	49.70	0.92
B_30	1502.24	0.96	7.02	0.74	675.47	63.46	0.96

## Data Availability

The original contributions presented in this study are included in the article/Supplementary Material. Further inquiries can be directed to the corresponding author.

## Supplementary Materials

The following supporting information can be downloaded at: https://www.mdpi.com/article/10.3390/toxics13050405/s1, Table S1. Physical and chemical properties of the experimental soils.
