# Supplementary material for: Biodegradation of Benzo(a)pyrene in Contaminated Soil: Plant and Microorganism Contributions from Isotope Tracing
Source: Toxics. 2025 May 16;13(5):405. doi: 10.3390/toxics13050405 (PMC12115903; doi:10.3390/toxics13050405)
Supplement: Supplementary file 1 [file toxics-13-00405-s001.zip › toxics-3608084-supplementary.pdf]

Supporting information for

Biodegradation of Benzo(a)pyrene in Contaminated Soil: Plant and Microorganism Contributions from Isotope Tracing

Jianlong Wang [1,2,*], Xiaobing Su [1,2], Changhe Zhang [1,3], Zhimeng Han [1] and Meiqi Wang [1]

[1] Key Laboratory of Urban Storm Water System and Water Environment, Ministry of Education, Beijing University of Civil Engineering and Architecture, Beijing 100044, China
[2] Beijing Energy Conservation & Sustainable Urban and Rural Development Provincial and Ministry Co-construction Collaboration Innovation Center, Beijing 100044, China
[3] China Academy of Building Research, Beijing 100013, China
* Correspondence: wangjianlong@bucea.edu.cn

Content of this file

Table S1. Physical and chemical properties of the experimental soils

Soil type	pH	Volumetric weight (g/cm^3)	Soil organic matter (g/kg)	TOC (g/kg)	TN (mg/kg)
Non-contaminated soil	9.03	1.27	22.27	2.32	668.44
contaminated soil	8.91	1.12	29.24	4.26	251.62
